# What Does the History of Research on the Repair of DNA Double-Strand Breaks Tell Us?—A Comprehensive Review of Human Radiosensitivity

**DOI:** 10.3390/ijms20215339

**Published:** 2019-10-26

**Authors:** Elise Berthel, Mélanie L. Ferlazzo, Clément Devic, Michel Bourguignon, Nicolas Foray

**Affiliations:** 1 Inserm, UA8 Unit, « Radiation: Defense, Health and Environment », Centre Léon-Bérard, 28, rue Laennec, 69008 Lyon, France ; Elise.berthel@inserm.fr (E.B.); Melanie.ferlazzo@inserm.fr (M.L.F.); clement.devic@inserm.fr (C.D.); Michel.bourguignon@irsn.fr (M.B.); 2 Université Paris Saclay (UVSQ), Versailles St Quentin en Yvelines, 78035 Versailles, France

**Keywords:** DNA double-strand breaks, DSB repair, non-homologous end-joining, radiation, radiosensitivity

## Abstract

Our understanding of the molecular and cellular response to ionizing radiation (IR) has progressed considerably. This is notably the case for the repair and signaling of DNA double-strand breaks (DSB) that, if unrepaired, can result in cell lethality, or if misrepaired, can cause cancer. However, through the different protocols, techniques, and cellular models used during the last four decades, the DSB repair kinetics and the relationship between cellular radiosensitivity and unrepaired DSB has varied drastically, moving from all-or-none phenomena to very complex mechanistic models. To date, personalized medicine has required a reliable evaluation of the IR-induced risks that have become a medical, scientific, and societal issue. However, the molecular bases of the individual response to IR are still unclear: there is a gap between the moderate radiosensitivity frequently observed in clinic but poorly investigated in the publications and the hyper-radiosensitivity of rare but well-characterized genetic diseases frequently cited in the mechanistic models. This paper makes a comprehensive review of semantic issues, correlations between cellular radiosensitivity and unrepaired DSB, shapes of DSB repair curves, and DSB repair biomarkers in order to propose a new vision of the individual response to IR that would be more coherent with clinical reality.

## 1. Introduction

The statement that radiation-induced damage inside the cell nucleus are the key-elements of the individual response to ionizing radiation (IR) is much anterior to the discovery of the DNA structure itself. Indeed, at the beginning of the 20th century, from pioneer observations on irradiated cells, Claudius Regaud showed that “the most radiosensitive component of the cell is the nucleus” [1,2]. Thereafter, radiobiological studies were essentially based on cytogenetic observations with the first assessments of the radiation-induced chromosome breaks. Since radiation-induced chromosome breaks may be generated by the propagation of radiation-induced DNA breaks throughout the cell cycle, cytogenetics research has been associated with DNA repair research since the discovery of the DNA structure in the 1950s. DNA double-strand breaks (DSB) progressively appeared to be the key-DNA damage responsible for cellular death if unrepaired and for cellular transformation if misrepaired [3]. This paper provides a comprehensive review of individual radiosensitivity and radiation-induced DSB repair and signaling all along the history for radiation research. It notably focuses on semantic issues, correlations between cellular radiosensitivity and unrepaired DSB, shapes of DSB repair curves, DSB repair biomarkers, and clinical data in order to propose a new vision of the individual response to IR.

## 2. What does Semantics Tell Us?

**Figure 1:**
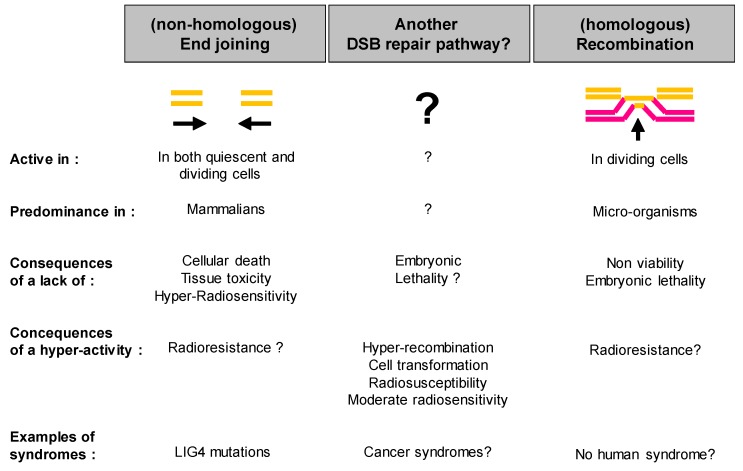
Schematic view of the DNA double-strand breaks (DSB) repair pathways and their clinical consequences in the case of defect or hyper-activity. The existence of another DSB repair pathway is discussed in the next chapters.

The first DSB repair pathway that was discovered, namely the recombination pathway, was pointed out with micro-organisms, whose major characteristics are a high rate of proliferation and a small genome length [4,5,6]. In other words, the first features of the cellular response to IR have been observed on duplicating (G2/M) cells rather than on quiescent (G0/G1) cells and in a reduced DNA sequence. Because of the close vicinity of homologous strands during mitosis, the recombination pathway was called homologous recombination (HR). HR is specifically activated in the G2/M phase. Hence, unlike in micro-organisms, HR is not predominant in mammalian cells since they are mostly quiescent [6] (Figure 1).

In the 1980s, a new DSB repair pathway, the DNA end-joining pathway, was discovered. The end-joining pathway is active in G0/G1 phase but also in G2/M phase. However, again, since mammalian cells are mostly quiescent while micro-organisms are generally proliferating cells, the end-joining pathway is predominant in mammalian cells and less frequent in micro-organisms [7,8]. Because the term “homologous recombination” was already extensively used by radiobiologists, the DNA end-joining pathway was called non-homologous end-joining (NHEJ) [8]. Nevertheless, it must be stressed here that the notion of DNA strand homology does not necessarily have the same meaning for a recombination and an end-joining process: recombination results in an exchange of DNA strands that can be homologous or non-homologous sequences, while an end-joining process consists of joining DNA ends independently of the DNA sequence [9]. The question this raises is: what would homologous end-joining mean? Despite this semantic contradiction, “non-homologous end-joining” has like “homologous recombination”, become a current term [10]. These two terms have contributed to propagating the false idea that there are only two repair pathways to manage DSBs: a homologous (HR) and a non-homologous (NHEJ) pathway, namely the “HR or else NHEJ paradigm” [3].

However, some cells elicit a significant radiosensitivity while showing both normal homologous recombination (HR) and non-homologous end-joining (NHEJ) pathways, thereby suggesting the existence of other DSB repair pathways [7,9]. Hence, at the end of the 1980s, further investigations about DSB repair and radiosensitivity were performed to verify the biological and clinical relevance of the “HR or else NHEJ paradigm” [3] (Figure 1).

## 3. What does Correlation Between Cellular Radiosensitivity and DSB Repair Tell Us?

**Figure 2:**
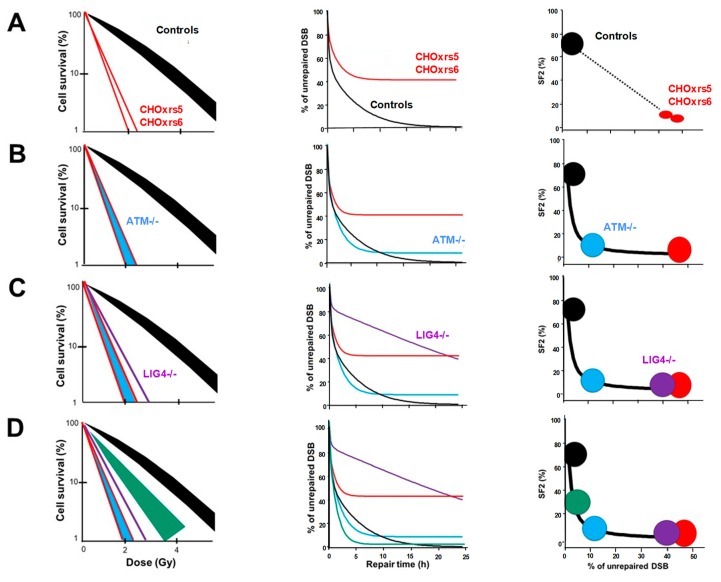
Brief history of the correlations between cellular radiosensitivity and unrepaired DSB through the cell survival curve and DSB repair kinetics obtained with pulsed field gel electrophoresis (PFGE) and relationships between cell survival at 2 Gy (SF2) and unrepaired DSB. All the data are provided from references [12,15,16]. Black, red, purple, and blue symbols and lines reflect the radioresistant cells, CHO xrs5 and xrs6, *LIG4*-mutated, *ATM*-mutated cells, respectively. The green symbols and lines represent representative cells with moderate radiosensitivity [12]. Panels (**A**–**D**) correspond to different steps in the history of DSB repair research (see the corresponding text).

In the 1980s, Fertil and Malaise showed that in vitro clonogenic cell survival is quantitatively correlated to the in vivo local tumour control [11]. To better define the molecular and cellular bases of individual radiosensitivity, radiobiologists have focused on the link between clonogenic cell survival and the yield of unrepaired DSB [3,12]. In the same period, by using the standard method of microbial genetics and applying a treatment of ethyl methanesulphonate to cells for 24 h, Jeggo and Kemp isolated 8 X-rays-sensitive mutants of Chinese Hamster Ovary (CHO) cells [13,14,15]. These cell lines were called CHO - x-rays sensitive (CHO-xrs). Among them, only the two most radiosensitive (xrs-5 et xrs-6) received focused research efforts. Molecular investigations showed that the xrs-5 and xrs-6 cell lines repaired only 50% of radiation-induced DSB and their corresponding in vitro cell survival fractions at 2 Gy (SF2) were about 1% [13,14,15]. In the radioresistant CHO controls, the DSB repair was complete and the SF2 reached 80%. These data led to the first correlation between a DSB repair defect and cellular radiosensitivity in mammalian cells (Figure 2A).

While the other CHO-xrs cell lines were neglected, further investigations pointed out that the hyper-radiosensitive xrs-5 and xrs-6 cell lines were both mutated for the Ku80 protein, an essential component of the DNA-PK kinase, a key-actor of NHEJ [17,18]. This conclusion strengthened the hypothesis that an impaired NHEJ was the major cause of radiosensitivity, whereas the HR contribution to human radiosensitivity, as defined as attributable to radiation-induced cellular death, is low [3]. Some other radiosensitive rodent cell lines were isolated by following the similar mutagenic treatment approach described above. However, again, the cell lines that did not elicit hyper-radiosensitivity were neglected [19]. Such a selection, together with the lack of moderate radiosensitivity data, appeared to be consistent with the hypothesis that radiosensitivity was an all-or-none phenomenon predominantly due to a lack of NHEJ functionality: it is the “all NHEJ paradigm”.

All the works described above were based on rodent cell lines. While the hyper-radiosensitivity of fibroblasts derived from ataxia telangiectasia (AT) patients was pointed out in 1975 [20], the first observations of DSB repair in AT cells led to the striking conclusion that DSB repair was normal [20,21,22,23,24]. However, these studies were limited to 4 h post-irradiation. Further investigations up to 24 h post-irradiation showed that AT cells repaired nearly 90% of the radiation-induced DSB. Their SF2 was similar to the most hyper-radiosensitive rodent cell lines [22] (Figure 2B). The differences between the DSB repair rate of the hyper-radiosensitive rodent and human cells needed to be explained. Some authors considered that it was not that the unrepaired DSB and NHEJ defects could not explain radiosensitivity in humans while successfully explaining radiosensitivity in rodents [25,26]: the “all NHEJ paradigm” appeared to be relevant in rodents but not in humans. In 1995, a young patient suffering from lymphoma succumbed to its radio-chemotherapeutic treatment without showing mutations in the *ATM* gene similar to those observed with the AT syndrome: it was later discovered that this young patient was suffering from a mutation of ligase IV (LIG4), which is essential for the NHEJ pathway [27,28,29]. The fibroblasts derived from this patient showed cellular radiosensitivity and DSB repair data similar to those of the CHO-xrs-5 and xrs-6 cell lines (Figure 2C). Thus, some authors considered that the unrepaired DSB might not explain radiosensitivity in AT cells while they might explain radiosensitivity in rodents and in *LIG4*-mutated human cells [26]. Hence, the “all NHEJ paradigm” appeared to be relevant in mammalians to the notable exception of the highest radiosensitivity observed in the AT syndrome [3] (Figure 2C).

**Figure 3:**
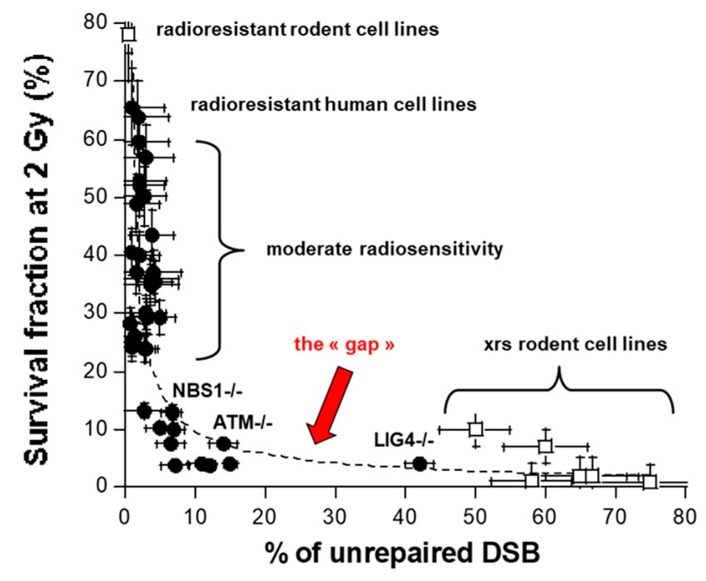
Correlation between survival fraction at 2 Gy and the percentage of unrepaired DSB for the xrs rodent cell lines and their radioresistant controls (data from [15] and human fibroblasts (data from reference [12] and reference [30]). The *ATM*, *NBS1,* and *LIG4*-mutated cells hold a specific label. The data obey either a power function (*y* = 55.36*x*^−0.76^; *r* = 0.68) or an inverse function (*y* = 75/(*x* + 0.57); *r* = 0.63) (dotted line).

In order to determine the actual nature of the correlation between DSB repair and radiosensitivity and to put an end to all these contradictions, a systematic study of survival and DSB repair data in a very large spectrum of human radiosensitivity became necessary. However, such a collection of human cells did not exist yet. In 2008, by analyzing about 40 human untransformed cell lines representing eight genetic diseases associated with radiosensitivity with a tens of different techniques, an inversed correlation was observed between cell survival and DSB repair: the higher the number of unrepaired DSB, the more radiosensitive the cell [12]. Furthermore, a continuum of cellular radiosensitivity level was observed, with SF2 values ranging from 1% to 70% in normal human cells (the SF2 of the most radioresistant rodent cell line and that of the most radioresistant human tumour cell line reach about 80%) [3]. By contrast, with regard to unrepaired DSB, a range of responses was observed from 0% of unrepaired DSB at 24 h post-irradiation (corresponding to radioresistance) to about 15% of unrepaired DSB at 24 h post-irradiation (corresponding to the hyper-radiosensitivity of AT cells) (Figure 2D). A gap in the percentage of unrepaired DSB between 15% to 35% was also observed, reflecting a discontinuity in the spectrum of the percentage of unrepaired DSB [12] (Figure 2 and Figure 3). Still to date, there is no example of a human normal cell line whose percentage of unrepaired DSB is higher than 15% and lower than 35%. Similarly to the NHEJ-defective rodent cell lines, the percentage of unrepaired DSB observed in *LIG4*-mutated cells ranges between 35% and 40% [27,28]. Hence, even if the inverse correlation between cellular radiosensitivity and DSB repair rate is consolidated by data from a number of genetic syndromes, the gap observed between 15% and 35% of unrepaired DSB raises a number of questions: is it specific to human cells? Does it depend on the DSB repair assay applied? Does it finally support the “all NHEJ paradigm”?

After plotting the survival data against the corresponding percentage of unrepaired DSB obtained from the xrs cell lines [15], a quantitative correlation appeared (Figure 3). Such a correlation was found compatible to that obtained with human cells and revealed that a gap is observed in the 15%–35% range of percentage of unrepaired DSB for both rodent and human cells (Figure 3). Hence, a large spectrum of radiation responses is a common feature of radiosensitivity in mammalians but some historical choice of the most radioresistant and hyper-radiosensitive rodent cells may have been the source of biases. The existence of a gap between the moderate radiosensitivity phenotype and a gross lack of NHEJ activity raised the question of potential artefacts linked to the technique used. At this step, a systematic review of DSB repair assays and DSB repair curves becomes necessary.

## 4. What Does the Shape of DSB Repair Curves Tell Us?

**Figure 4:**
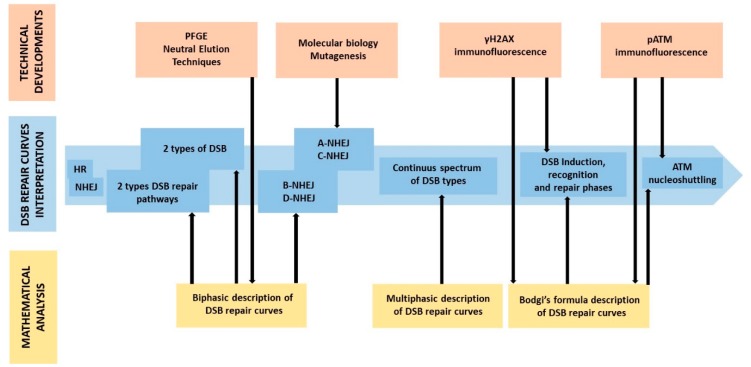
Evolution of the interpretation of the DSB repair curves. Schematic representation of the different mechanistic and mathematical models for describing DSB repair curves. The Bodgi’s formula refers to a single formula used to described kinetics of nuclear foci observed with immunofluorescence [36].

The first experimental approaches developed to assess DSB, namely the sucrose gradient sedimentation, the neutral elution, and the pulsed field gel electrophoresis (PFGE) techniques [31], were based on the discrimination of the radiation-induced DNA fragments by their size. In fact, immediately after irradiation, DNA is fragmented by the DSB induction that obeys the following rule: the higher the dose, the higher the number of DNA fragments and the lower their size. Furthermore, during the DSB repair, the number of DNA fragments decreases and their average size increases [32]. A technique based on the discrimination of the radiation-induced DNA fragments is useful for assessing the DSB repair rate, independently from the different DSB repair pathways activated. Because these assays were limited to the detection of long DNA fragments (more than 15 Mbp), they required high radiation doses (some tens of Gy) that were not biologically relevant [31]. Despite such potential bias, when data are expressed as a percentage of unrepaired DSB as a function of post-irradiation repair time, the shape of DSB repair curves does not change drastically with the initial dose [33]. Furthermore, when data are plotted in a semi-log scale, the DSB repair curves obtained with these techniques appear to be biphasic with a “fast” component corresponding to a repair half-time of several minutes and a ”slow” component corresponding to repair half-time of several hours [31]. Since the 1980s, such a biphasic shape of curves was interpreted by the existence of two types of DSB repaired by a unique DSB repair pathway [34] or the existence of two independent DSB repair pathways acting at two different rates on a unique population of DSB [35]. However, the biexponential formula is too mathematically flexible to distinguish what model (two types of DSB or two DSB repair pathways?) is the best one. Besides, there was no further hypothesis about the actual nature of these pathways and the NHEJ and HR pathways were not necessarily cited to explain the biphasic shape of DSB repair curves until the 2000s [31] (Figure 4).

More recently, such biphasic description was at the basis of the hypothesis of the backup/alternative NHEJ pathways [37,38]. Indeed, when one of the component of the trimeric DNA-PK kinase (Ku70, Ku80 and DNA-PKcs) is mutated, or when LIG4 is mutated, the “fast” component contribution decreases drastically, which has been interpreted as a reduced activity of a DNA-PK-dependent NHEJ pathway (D-NHEJ). However, the inhibition of the DNA-PK activity never leads to a total deactivation of DSB repair but rather to residual DSB repair activity called “backup” NHEJ (B-NHEJ), likely characterized by the slow repair component. The “B- and D-NHEJ” model was proposed by G. Iliakis’s group from 2000 and was initially based on the biphasic description of the DSB repair curves [37,38,39,40,41,42] (Figure 4).

Some years later, from mutagenesis or genetic investigations, and independently of the biphasic description of the DSB repair curves, other models of NHEJ variants have been hypothesized and were described as “alternative” NHEJ pathway (A-NHEJ) or as a “classical” or “canonical” NHEJ pathway (C-NHEJ). However, this “A- and C-NHEJ” model is still not consensual and a number of different A-NHEJ flourished. This is notably the case of an ATM- and NBS1-dependent switch recombination pathway [43], a PARP1-, LIG3-, and XRCC1-dependent end-joining pathway [44], a BLM-dependent but LIG4-independent end-joining pathway [45], a micro-homology associated end-joining pathway [46], and a MRE11-dependent DSB repair pathway [47], while also being associated with DNA ligase III [48]. All these different models have contributed to increasing the confusion about the description of the A-NHEJ pathway, although they show some common features like the presence of short tracts of sequence homology (microhomologies) at repair sites and chromosome translocations, suggesting an implication in the carcinogenesis process [48]. Hence, some A-NHEJ pathways are often considered as a microhomology end-joining or a single-strand annealing pathway [49]. Furthermore, by adding an extra-complexity to our vision of DSB repair, the A-NHEJ pathway is not necessarily equivalent to the B-NHEJ pathway evoked above, while C-NHEJ is likely to be DNA-PK- and LIG4-dependent and refers to D-NHEJ. Nevertheless, all these investigations and hypotheses converge toward the same conclusion: in addition to the NHEJ and the HR, an alternative DSB repair pathway should exist, be active in G0/G1 cells, and be responsible for genomic instability and cancer proneness (Figure 1). In this frame, both the “NHEJ or else HR paradigm” and “all NHEJ paradigm” appear to be irrelevant (Figure 4).

In parallel to these numerous molecular models, a deeper mathematical kinetic analysis of the DSB repair curves showed that their shape is more multiphasic than biphasic [50,51]. Indeed, the values of the two repair half-times strongly depend on the maximal post-irradiation repair time investigated [31,51]. In other terms, the description of the DSB repair curves is similar to the problem of the quadrature of the circle interpretation (how to describe a curve with straight portions?). Hence, a multiphasic model was shown to provide a better fit of repair data than a biphasic one [51]. Consequently, the shape of the DSB repair curves can be interpreted as a result of a continuous and multiexponential process and a large spectrum of DSB characterized by their own repair half-time, whatever the DSB repair pathway. As the repair time increases, DSB are repaired and disappear progressively in line with their inherent repair-half time. As a result, the number of DSB decreases but the average of the inherent repair-half time at a given repair time increases [51]. Such a model strongly supports the diversity of the DSB severity. But a question remains: how should we further investigate the different DSB repair pathways? (Figure 4).

## 5. What do the New DSB Biomarkers Tell Us?

**Figure 5:**
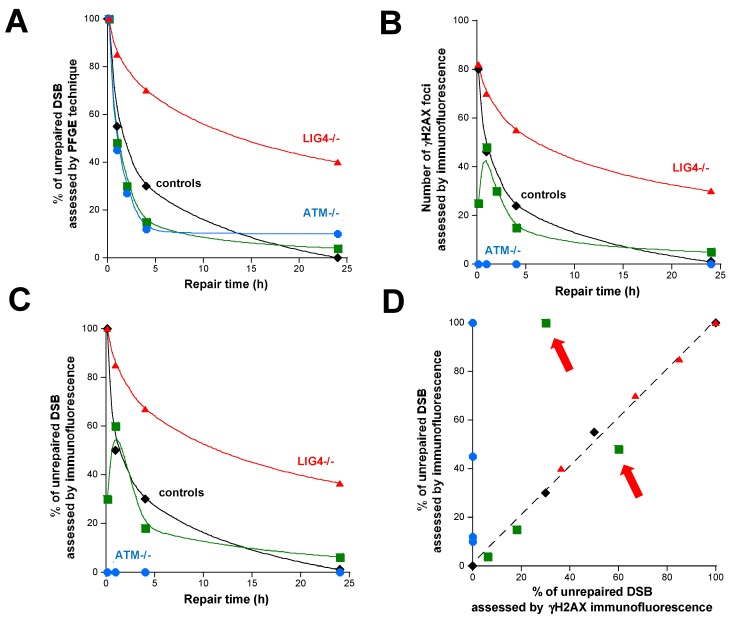
A comparison between DSB data obtained from the pulsed field gel electrophoresis (PFGE) technique and γH2AX immunofluorescence. The DSB repair data are provided from reference [12] for PFGE (**A**) and reference [16] for γH2AX immunofluorescence (as absolute number of foci (**B**) or as percentage of remaining foci (**C**)). Data were fitted to a multiphasic model described in reference [12] and the Bodgi’s formula described in references [16,36]. For convenience, error bars were omitted. Like in Figure 2, the green plots represent the data provided from representative human cells showing moderate radiosensitivity like certain cases of Fanconi anaemia or Bloom’s syndrome [12]. In panel (**D**), the dotted line represents a linear fitting formula (*y* = *x* + 1; *r* = 0.99). The red arrows represent the early data plots of cells with moderate radiosensitivity that are not in agreement with the linear formula together with the data from the *ATM*-mutated cells (blue plots).

In the 2000s, the immunofluorescence technique appeared that permits us to assess the subcellular localization of a given protein after irradiation via specific antibodies [52,53,54,55]. Among the numerous proteins involved in the DSB repair pathways, several tens localize as nuclear foci after irradiation, which allows their quantification as a function of dose and time [36]. However, it must be stressed that the formation of nuclear foci by a given protein corresponds to various steps of the DSB repair process that need to be identified. Among them, the phosphorylated form of the variant histone H2AX (γH2AX) holds a special place in the DSB repair pathway since a one-to-one correlation was obtained between the number of radiation-induced DSB and the number of nuclear γH2AX foci [56]. The γH2AX assay drastically decreases the lower limit of radiation dose to be investigated and offers a powerful tool to study low-dose phenomena in the mGy range [56]. However, historically, the first one-to-one correlation described between DSB and γH2AX foci was only verified with one radioresistant and the hyper-radiosensitive *LIG4*-mutated fibroblast 180BR cell line [56]. For example, the hyper-radiosensitive *ATM*-mutated cells show very few γH2AX foci, if any, whatever the post-irradiation time. Conversely, the hyper-radiosensitive *DNA-PK* or *LIG4*-mutated cells show normal yields of γH2AX foci some minutes after irradiation and significant residual DSB 24 h after irradiation, suggesting that ATM but not DNA-PK or LIG4 is required for an early radiation-induced phosphorylation of H2AX [12,57,58]. More recently, one hundred fibroblast cell lines from patients showing moderate radiosensitivity were found to elicit an abnormally low number of early γH2AX foci. Hence, the shape of DSB repair curves obtained with PFGE and γH2AX immunofluorescence may be different according to the cell lines [36,59] (Figure 5). A more rigorous analysis of the DSB repair curves obtained with γH2AX immunofluorescence was therefore needed.

By using immunofluorescence, some proteins relocalize as nuclear foci in response to IR in two kinetic phases: 1) the foci appearance phase during which the number of foci increases and reaches its maximum. This phase can be considered as a DNA damage recognition phase; 2) the foci disappearance phase during which the number of foci decreases. Such a phase can be considered as the result of the repairing of DNA damage [36]. At this stage, it must be stressed that the immunofluorescence assays are applied to biologically and clinically relevant doses (0–2 Gy) while the initial doses required by the DSB assays based on the discrimination of the radiation-induced DNA fragments were more than 10 times higher, at least. Hence, it is likely that very high doses can stimulate the DSB repair and signaling system so much that the DNA damage recognition phase becomes very fast and is not identifiable on the DSB repair curves (Figure 5).

The diversity of immunofluorescence biomarkers that formed nuclear foci has not facilitated the elucidation of consensual mechanisms of DSB repair: to date, nearly all the foci-making proteins were found to co-localize. This is notably the case for γH2AX and MRE11 [60], γH2AX and 53BP1 [61], or γH2AX and DNA-PK [12]. However, in immunofluorescence technique, the emission and/or absorption spectra of immunofluorescence dyes (often red and green) are not strictly separated, which causes biased co-localization (the bleed-through phenomenon) [60]. Hence, a number of authors who have researched the co-localization of two biomarkers have not verified whether the co-immunofluorescence data were consistent with the foci kinetics obtained from separated immunofluorescence. Furthermore, co-localization is often observed on specific cell lines, at a given post-irradiation time, and after a given dose. Hence, the protein partnership and the mechanistic models that are deduced from it may be biased by the bleed-through phenomenon whose occurrence is clearly independent of any confocal or non-confocal technology. Hence, particular care must be taken with co-immunofluorescence data and the resulting mechanistic models [60].

## 6. What do the Clinical Data Teach Us?

As discussed above, the great majority of the radiobiological investigations have been done with very radioresistant and hyper-radiosensitive cells. However, the hyper-radiosensitive and hyper-radioresistant cellular models are not representative of the clinical reality. For example, there is no known human genetic syndromes caused by mutations of Ku or DNA-PKcs proteins, the major NHEJ actors. The only human case of gross defect in NHEJ function to non-viability was caused by mutations of DNA ligase IV (LIG4) [29,62]. The majority of cases of fatal post-radiotherapy events were encountered after whole-body irradiation of children with ataxia telangiectasia (AT) caused by homozygous *ATM* mutations during their anti-leukemia or lymphoma treatment [63,64,65,66]. It is important to note that AT frequency is of about 1/100,000. By contrast, about 5%–20% cancer of patients treated by radiotherapy exhibit post-radiotherapy tissue reactions that can be considered as moderate radiosensitivity. Surprisingly, no or very few rodent cells with moderate radiosensitivity have been the subject of a published radiobiological characterization [66]. Interestingly, some of the proteins whose mutations are responsible for these syndromes were cited as components of the different variants of A-NHEJ (see Section 4). In addition, some genetic syndromes, associated or not with cancer proneness, different from the precited ones, also show a moderate radiosensitivity: this is notably the case of neurofibromatosis [67], Huntington’s chorea [68], and tuberous sclerosis syndromes [30]. It must be stressed that these syndromes are caused by mutations of cytoplasmic proteins, which may contradict the historical hypothesis that nucleus is the most radiosensitive part of the cell (see the Introduction). Furthermore, it also contradicts the hypothesis that defect of DSB repair is the unique cause of cellular radiosensitivity. The question of how to build a unified model that would describe both hyper-radiosensitivity observed in *LIG4*- and *ATM*-mutated cells and moderate radiosensitivity caused by impairment of alternative signaling and DSB repair pathways remains.

**Figure 6:**
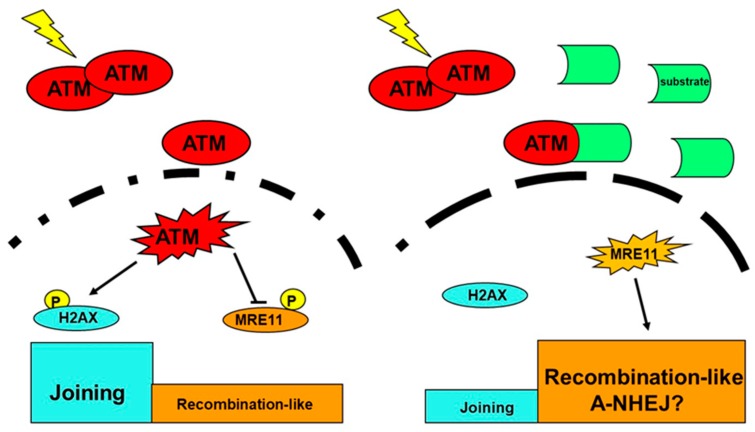
Schematic representation of the radiation-induced ATM nucleoshuttling (RIANS) model. Left panel: Radioresistance. After irradiation, the radiation-induced ATM monomers diffuse in nucleus and phosphorylate H2AX at the DSB sites, which activates NHEJ, while the ATM phosphorylation of other actors involved in alternative pathways can be inhibited. Right panel: Moderate radiosensitivity. Some over-expressed ATM substrates sequestrate ATM monomers in cytoplasm which prevents or delays RIANS and activation of NHEJ. In the meantime, alternative DSB repair pathways increase cancer or aging risk. The different dashed lines illustrate the hypothesis that the permeability of the nuclear membrane may be different in the radioresistant and radiosensitive cases [72].

To date, the ATM protein has been considered as an early actor of the DSB recognition via its phosphorylation of H2AX and the individual radiation response [69,70]. Surprisingly, ATM has not been integrated in all the DSB repair models discussed above while mutations of RAD51 and RAD52 (for HR) and Ku and DNA-PKcs (for NHEJ) do not cause any viable syndromes in humans (Figure 1). Recently, the delay in the radiation-induced nucleo-shuttling of ATM protein (RIANS) was shown to be a reliable parameter for predicting radiosensitivity [36,59,68] and to provide a biologically relevant interpretation of the linear-quadratic model, the mathematical basis of the cellular radiation response [71]. In the context of the RIANS model, the following mechanistic hypothesis has been proposed: after irradiation, cytoplasmic trans-auto-phosphorylated dimeric forms of ATM become monomeric and diffuse to the nucleus. In the nucleus, active ATM monomers phosphorylate H2AX molecules at DSB sites, which activates NHEJ. The ATM monomers will re-dimerize during the DSB repair process. Once in nucleus, ATM may also inhibit MRE11 nuclease activity or any other actor responsible for genomic instability. By contrast, in cells with moderate radiosensitivity, over-expressed ATM substrates may delay the RIANS, thereby favoring cancer proneness and aging. This is notably the case for huntingtin, neurofibromin, and tuberin [36,68,72]. (Figure 6).

Three groups of radiosensitivity have been defined [59,71,72,73,74,75,76]:

Group I (about 75% to 85% whole population) represents the normosensitive (radioresistant) patients with a rapid RIANS after 2 Gy, a low risk of post-radiotherapy tissue reaction and of cancer

Group II (about 5–20% of whole population) represents the patients who elicit a moderate radiosensitivity with a delay in the RIANS due to the sequestration of ATM by mutated and over-expressed substrates. These patients are moderately radiosensitive and may have a high risk of cancer or neurodegeneration.

Group III (<1% whole population) represents the *ATM*-mutated patients with no functional ATM kinase or those who show strong DSB repair defects (like the *LIG4*-mutated patient described above), hyper-radiosensitivity, and either high cancer proneness or severe accelerated aging [12,73].

While it provides relevant explanation of the radiosensitivity of syndromes caused by mutations of cytoplasmic proteins, the RIANS model appears to be compatible with all the alternative models, as ATM is upstream of all the cascades of radiation-induced phosphorylations involved in the individual response to IR [77]. Obviously, further investigations are needed to better understand the direct or indirect role of cytoplasmic ATM forms in the activation or the inhibition of DSB repair pathways.

## 7. Conclusions

At the beginning of the 20th century, radiobiologists believed that individual response to IR was dependent on the DNA damage induced in the nucleus alone. To date, radiosensitivity also appeared to be caused by mutated cytoplasmic proteins. At the end of the 1980s, the “NHEJ or else paradigm” supported the theory that only two pathways were required to repair DSB. To date, by focusing on clinical reality, semantic issues, and technical artefacts linked to DSB assays or cellular models, evidence can be found suggesting that the individual response to IR is complex and that deciphering the DSB repair mechanisms needs more rigorous methodology and a constant link towards clinicians. To better quantify the radiation-induced risks, a general survey of human radiosensitivity from clinical and not molecular data is required to identify the proteins involved in DSB repair in realistic conditions of medical exposure to IR.
